# 

**Published:** 1893-09-16

**Authors:** 


### SPECIAL STUDENTS' NUMBER.

### CONTENTS.

**Metropolitan Medical Schools and their Common Interests** - 385

**The Late Surgeon-Major Parke** - 386

**From Chemist's Shop to Physic's Throne** - 387

**The Cost of a Medical Education** - 388

**How a Lady may Become a Doctor** - 389

**Annotations.**—Reckless Cycling.—How to "Dish" the Food Adulterator.—Wanted, a Medical Defoe - 391

**Notes and News** - 392

**The Medical Student's Vade Mecum**—

METROPOLITAN MEDICAL SCHOOLS - 393

DENTAL SCHOOLS IN LONDON - 400

MEDICAL EDUCATION IN SCOTLAND - ix

THE BOOK WORLD FOR MEDICAL STUDENTS - xi

**The Editor's Letter-Box** - 399

**New Appliances and Things Medical** - 399

### EXTRA SUPPLEMENT.—THE NURSING MIRROR.

**News from the Nursing World.**—Our Needlework Competition.—Intelligent Precautions.—At Less than Cost Price.—Much too Young.—People who "Want to Know."—Grateful Nurses.—An Important Decisioo.—At Home and Abroad.—English Nurses in Paris.—Gifts Easy to Choose.—Short Items - ccxli
**Trained Nursing in Berlin** - ccxliii
**The Position of Nurses.**—By A NURSE - ccxliv
**Wants and Workers** - ccxlvi
**Nursing in South Africa** - ccxlv
**Every-day Dangers.**—Nursing in a Cottage Hospital - ccxlv
**Everybody's Opinion.**—A Correction—District Nursing—An Excellent Defence.—An Unprofessional Correspondent - ccxlvi
**Notes and Queries** - ccxlvii
**For Reading to the Sick.**—Prayer—III. - ccxlvii
**Death in Our Ranks—Our Examinations** - ccxlvii
**Appointments** - ccxlvii
**The Muses' Looking Glass.**—Some Thoughts on the Importance of Early Nursery Training - ccxlviii

